# Subacromial impingement syndrome and pain: protocol for a randomised controlled trial of exercise and corticosteroid injection (the SUPPORT trial)

**DOI:** 10.1186/1471-2474-15-81

**Published:** 2014-03-14

**Authors:** Edward Roddy, Irena Zwierska, Elaine M Hay, Sue Jowett, Martyn Lewis, Kay Stevenson, Danielle van der Windt, Nadine E Foster

**Affiliations:** 1 Arthritis Research UK Primary Care Centre, Research Institute for Primary Care and Health Sciences, Keele University, Keele, Staffordshire ST5 5BG, UK; 2 Staffordshire Rheumatology Centre, Haywood Hospital, High Lane, Burslem, Stoke-on-Trent ST6 7AG, UK; 3 Physiotherapy Department, University Hospital of North Staffordshire, Stoke-on-Trent, UK

**Keywords:** Subacromial impingement syndrome, Shoulder pain, Randomised controlled trial, Corticosteroid injection, Musculoskeletal ultrasound, Exercise, Physiotherapy

## Abstract

**Background:**

Subacromial impingement syndrome is the most frequent cause of shoulder problems which themselves affect 1 in 3 adults. Management commonly includes exercise and corticosteroid injection. However, the few existing trials of exercise or corticosteroid injection for subacromial impingement syndrome are mostly small, of poor quality, and focus only on short-term results. Exercise packages tend to be standardised rather than individualised and progressed. There has been much recent interest in improving outcome from corticosteroid injections by using musculoskeletal ultrasound to guide injections. However, there are no high-quality trials comparing ultrasound-guided and blind corticosteroid injection in subacromial impingement syndrome. This trial will investigate how to optimise the outcome of subacromial impingement syndrome from exercise (standardised advice and information leaflet versus physiotherapist-led exercise) and from subacromial corticosteroid injection (blind versus ultrasound-guided), and provide long-term follow-up data on clinical and cost-effectiveness.

**Methods/Design:**

The study design is a 2x2 factorial randomised controlled trial. 252 adults with subacromial impingement syndrome will be recruited from two musculoskeletal Clinical Assessment and Treatment Services at the primary-secondary care interface in Staffordshire, UK. Participants will be randomised on a 1:1:1:1 basis to one of four treatment groups: (1) ultrasound-guided subacromial corticosteroid injection and a physiotherapist-led exercise programme, (2) ultrasound-guided subacromial corticosteroid injection and an advice and exercise leaflet, (3) blind subacromial corticosteroid injection and a physiotherapist-led exercise programme, or (4) blind subacromial corticosteroid injection and an advice and exercise leaflet. The primary intention-to-treat analysis will be the mean differences in Shoulder Pain and Disability Index (SPADI) scores at 6 weeks for the comparison between injection interventions and at 6 months for the comparison between exercise interventions. Although independence of treatment effects is assumed, the magnitude of any interaction effect will be examined (but is not intended for the main analyses). Secondary outcomes will include comparison of long-term outcomes (12 months) and cost-effectiveness. A secondary per protocol analysis will also be performed.

**Discussion:**

This protocol paper presents detail of the rationale, design, methods and operational aspects of the SUPPORT trial.

**Trial registration:**

Current controlled trials ISRCTN42399123.

## Background

Painful shoulder problems are a common cause of impaired function and affect 1 in 3 adults [1,2], accounting for 1% of general practice (GP) consultations [3]. Subacromial impingement syndrome (SIS) is the most common cause of shoulder pain and affects approximately one-half of all shoulder pain sufferers [3]. SIS typically presents with pain during elevation of the arm, when a reduction in the space between the coracoacromial arch of the scapula and the humerus causes pinching of the subacromial/subdeltoid bursae, rotator cuff tendons and long head of biceps [4]. SIS is thought to arise as a result of bony abnormalities, rotator cuff weakness, impaired scapulohumeral rhythm, scapular instability and poor posture [4,5]. Treatment aims to reduce pain and increase function and UK management guidelines recommend exercise and corticosteroid injection in addition to patient education, oral analgesia and application of ice-packs [4,6]. In our recent observational study of patient outcome from treatment in primary-secondary care interface musculoskeletal clinics, 46% of patients with SIS received a corticosteroid injection and 38% were referred to a physiotherapist [7].

Exercise aims to reduce pain and functional impairment by improving posture, muscle strength, scapular stability and scapulohumeral rhythm [8]. It can be individualised, progressed and supervised by physiotherapists or self-guided from a leaflet containing standardised exercises [9]. Recent systematic reviews of exercise in SIS concluded that exercise decreases pain and improves function but that most trials are small, of poor quality, focus on short-term results only, and provide insufficient detail to allow firm conclusions to be drawn about the type, intensity, frequency and duration of exercise which is associated with best outcomes [10,11]. Consensus-based clinical guidelines recommend a ‘core’ set of exercises for SIS [4]. Studies in other musculoskeletal conditions support individualised, supervised and progressed exercise rather than a standard exercise approach [12] but, to date, there are few trials to guide exercise practice for patients with SIS [13-15].

Corticosteroid injections are commonly used to reduce pain and inflammation associated with SIS [16] although there is debate about their efficacy. Previous trials suggest similar effectiveness of corticosteroid injection and physiotherapy for both mixed shoulder problems [17,18] and SIS [19] whereas other trials report short-term superiority of injection over physiotherapy for mixed shoulder problems which is lost in the long-term [20,21]. A recent trial compared subacromial corticosteroid injection combined with exercise and manual therapy versus exercise and manual therapy alone for SIS and found similar improvements in pain and function at 3 months, however, more rapid reduction in pain and disability was seen in the group that received an injection [14]. These trials all used ‘blind’ shoulder injection, where the site of injection is located by observation and palpation. Poor response to blind injection has been attributed to misplaced injection [22-24].

Musculoskeletal ultrasound (MSUS) has become an increasingly accepted tool for guidance of therapeutic interventions [25,26]. However, although MSUS is low cost, can be carried out as a ‘bed-side’ procedure and has high resolution [26], few studies of ultrasound-guided (US-guided) injection for shoulder pain have been conducted. A recent RCT compared US-guided subacromial corticosteroid injection with systemic corticosteroid injection in patients with rotator cuff disease and found no difference in shoulder pain and function between groups at six weeks [27]. US-guided corticosteroid injection is suggested to provide greater improvements in pain, function and range of movement than blind injection for the treatment of SIS [28-30]. However, existing studies are small, of short duration, or are non-randomised, and none have originated from the UK [24,28-31].

The evidence supports the need for a high quality trial to determine how to optimise treatments for SIS patients and to reflect the complementary ways in which these interventions are used clinically i.e. that exercise and injection are often combined within the same package of care. The primary objectives of the SUPPORT trial (SUbacromial imPingement syndrome and Pain: a randomised controlled trial Of exeRcise and injecTion) are to assess whether (1) a physiotherapist-led intervention consisting of individualised, supervised and progressed exercise provides better improvements in pain and function than a standardised advice and exercise leaflet, and (2) US-guided subacromial corticosteroid injection provides better improvements in pain and function than unguided (blind) injection. Secondary objectives are to determine whether there is an interaction effect of combining US-guided injection and an individualised, supervised and progressed exercise programme and to conduct two separate cost-effectiveness analyses for the comparisons outlined in (1) and (2).

## Methods/Design

### Study design

**Figure 1:**
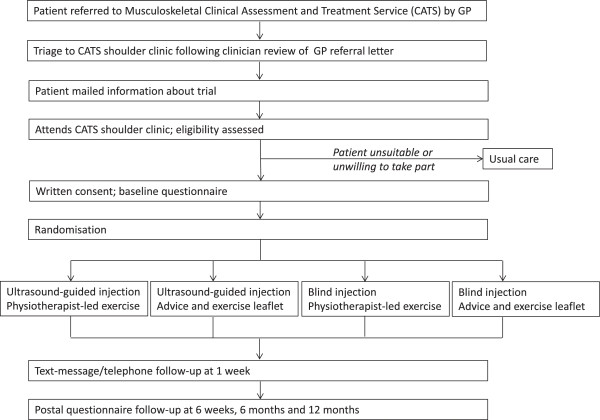
Flow of participants through trial.

The study will be a 2×2 factorial randomised controlled trial (Figure 1). Ethical approval has been obtained from The Black Country Research Ethics Committee (10/H1202/72).

### Setting

Patients will be recruited through musculoskeletal interface clinics in Staffordshire and Stoke-on-Trent Partnership Trust. These services incorporate early referral to, and rapid assessment by, specialist clinicians (rheumatologists, rehabilitation medicine specialists, extended scope physiotherapists and general practitioners with a special interest (GPwSI)) within a multidisciplinary team-working environment. Local family practices will be informed about the trial.

### Participants

Consecutive patients referred from primary care to interface shoulder clinics will be assessed for eligibility by interface clinicians.

#### Inclusion criteria

•18 years and over

•No history of significant shoulder trauma, for example, fracture, clinically-suspected full thickness cuff tear

•A clinical diagnosis of SIS (pain in deltoid insertion area, positive Neer and Hawkins-Kennedy tests, pain on shoulder abduction).

Accurate diagnosis of SIS is challenging but combination of the patients’ history and response to Neer and Hawkins-Kennedy tests (to rule SIS out with a negative test) and pain on shoulder abduction (to rule SIS in with a positive test) provides optimal sensitivity and specificity [5]. Patients will not be required to undergo diagnostic imaging (eg MRI) to reflect current practice where treatment choices are informed by clinical findings.

#### Exclusion criteria

•Below 18 years old

•Those whose main complaint is due to neck problems, acromioclavicular pathology, or other primary shoulder disorders including adhesive capsulitis or clinically-suspected full thickness cuff tear

•Potentially serious pathology (inflammatory arthritis, polymyalgia rheumatica, malignancy etc) or ipsilateral shoulder surgery/replacement

•Those already on a surgical waiting list for shoulder surgery

•Contra-indications to local corticosteroid injection (known blood coagulation disorders, warfarin therapy)

•Participation in a shoulder-focused exercise programme or shoulder injection in the last month

•Inability to provide informed consent, complete written questionnaires, or read instruction leaflets written in English

### Initial contact with participants

All patients referred to musculoskeletal interface services at each site with shoulder pain, identified from the referral letter from primary care, will be triaged to an interface service shoulder clinic. One to two weeks prior to their appointment in the interface service shoulder clinic all patients will be sent a letter of invitation and a Participant Information Sheet (PIS).

### Interface service shoulder clinic

Patients will attend for their routine clinic appointment, according to normal NHS clinical attendance procedures. The interface clinician will undertake the clinical consultation according to usual practice. Patients who fulfil the criteria for a diagnosis of SIS will be asked by the interface clinician if they wish to be considered for trial participation. Patients who are interested in trial participation will be invited by the interface clinician to see a Research Nurse, who will further confirm eligibility and explain the trial in full. Ineligible patients and those who do not wish to participate in the trial will receive normal clinical care delivered by the interface clinician according to usual practice. Anonymised age and gender will be collected for those patients who decline to take part, in order to assess the external validity of the recruited sample of patients.

Eligible patients who are interested in the trial will be asked by the Research Nurse to provide written informed consent to participate. The Research Nurse will allocate the participant a unique study number. The participant will then complete a baseline questionnaire (Table 1). The Research Nurse will check the baseline questionnaire for completeness, before the participant is randomised.

**Table 1:** Content of trial questionnaires

**Item**	**Baseline**	**6 weeks**	**6 months**	**12 months**
**Shoulder problem**				
Shoulder pain severity today (0-10 NRS)	X	X	X	X
Global change [20]		X	X	X
Potential adverse events		X		
Side of shoulder problem (right, left, both)	X			
Duration of shoulder problem	X			
History of previous shoulder problems	X			
Handedness (right or left)	X			
Shoulder Pain and Disability Index (SPADI) [32]	X	X	X	X
Effect of shoulder disability on typical everyday activities	X	X	X	X
Shoulder pain at night [33]	X	X	X	X
**Psychological factors**				
Brief illness perception questionnaire [34]	X	X	X	X
Pain self-efficacy questionnaire [35]	X	X	X	X
Tampa scale of kinesophobia [36]	X	X	X	X
**General health**				
EURO-QOL (EQ5D) [37]	X	X	X	X
MOS-Short Form 12 (SF-12) [38]	X	X	X	X
**Other health problems**				
Pain manikin	X	X	X	X
Co-morbidities	X			
**Treatment experiences, preferences, expectations**				
Previous experience of treatment	X			
Treatment preferences	X			
Expectations about different treatments	X			
Exercise adherence		X	X	X
**Confidence and satisfaction with treatment**				
Confidence in treatment		X	X	X
Treatment satisfaction		X	X	X
**Health-care utilisation**				
Consultation in primary and secondary care		X	X	X
Medication use (prescribed and over-the-counter)		X	X	X
Medical investigations		X	X	X
Hospital admission		X	X	X
**Effect of shoulder problem on work**				
Current/most recent job title and nature of work	X			
Current employment status	X	X	X	X
Work status including alteration in hours/duties	X			
Work absence	X	X	X	X
Work performance	X	X	X	X
Stanford presenteeism scale (SPS-6) [39]	X	X	X	X
Receipt of benefits, if not working	X	X	X	X
**Demographics**				
Age, gender	X	X	X	X
Living arrangements	X			
Height, weight	X			
Smoking status	X			

### Randomisation

As the Research Nurse will remain blinded to treatment allocation, a trial administrator in the interface service shoulder clinic will organise the randomisation, using Keele University’s Clinical Trial Unit’s telephone randomisation service, and then inform participants of their treatment allocation. Participants will be randomly allocated in a 1:1:1:1 ratio to 1 of 4 treatment groups using block randomisation to ensure that participants from each interface clinic have an equal chance of receiving any of the interventions:

•US-guided subacromial corticosteroid injection and physiotherapist-led individualised, supervised and progressed exercise

•US-guided subacromial corticosteroid injection and advice and exercise leaflet

•Unguided (blind) subacromial corticosteroid injection and physiotherapist-led individualised, supervised and progressed exercise

•Unguided (blind) subacromial corticosteroid injection and advice and exercise leaflet

The injection will be delivered in the same interface service shoulder clinic. Those randomised to receive the advice and exercise leaflet will receive this immediately following their injection. For participants randomised to receive the physiotherapist-led individualised, supervised and progressed exercise programme, the trial administrator will arrange their first physiotherapy appointment within 3 weeks of the shoulder injection, thereby meeting recommendations that recommendations that resistance exercise should be avoided for 2 weeks following steroid injection [4].

An evaluation of the success of Research Nurse blinding procedures will be completed and a procedure for reporting incidents where blinding has been compromised will be in place, through case report forms.

### Interventions

#### US-guided subacromial corticosteroid injection

Clinicians within the interface service shoulder clinics will deliver US-guided subacromial injection using a standard technique. Interface clinicians performing US-guided injections will comprise three rheumatologists, three extended scope physiotherapists, one musculoskeletal sonographer, one GPwSI, and one senior rheumatology nurse. Ultrasound examinations will be performed using the LOGIQe system with a 12 MHz transducer. The skin and transducer will be cleaned with chlorhexidine 0.5% solution and sterile gel will be applied to the transducer. The participant will be seated with the shoulder internally rotated and the ipsilateral hand placed on the buttock to allow greatest access to the subacromial bursa. The transducer will be placed anterolaterally and the hypoechoic subacromial bursa visualised. A 21G needle will be inserted under real-time US control until the needle-tip is seen to enter the bursa. A commercially available pre-mixed solution of methylprednisolone 40 mg and 1% lidocaine 1 ml will be injected in accordance with recent best-practice recommendations [4].

#### Unguided (blind) subacromial corticosteroid injection

Different interface clinicians to those providing the US-guided subacromial injections will deliver unguided (blind) subacromial injection using a standard technique. Unguided (blind) injectors will comprise two rheumatologists, one rehabilitation medicine specialist, four extended scope physiotherapists and a GPwSI. The participant will be seated with their arms hanging their by sides with elbow bent and forearm resting on lap. The skin will be cleaned with chlorhexidine solution 0.5%. A 21G needle will be inserted through the deltoid under the acromium process laterally and the same pre-mixed solution of methylprednisolone 40 mg and 1% lidocaine 1 ml will be injected.

Participants receiving either US-guided or unguided (blind) subacromial injection will be advised not to drive immediately after receiving the injection and to observe a period of relative rest for the following two weeks, avoiding pushing or pulling movements with the affected arm and heavy or repetitive tasks. Protocols for both US-guided and unguided injection will allow the injection to be repeated at the discretion of the treating interface clinician in event of partial response. In line with recommendations, a maximum of 2 injections will be permitted during follow-up per participant [4], and the number of injections received will be recorded.

#### Physiotherapist-led individualised, supervised and progressed exercise

The individualised, supervised and progressed exercise programme will be delivered by community-based, musculoskeletal physiotherapists and commenced within 3 weeks of injection. Exercise type, dose and progression will be individualised, supervised and progressed in 6 to 8 sessions over a 12 to 16 weeks. Based on available guidelines [4], a survey of local senior clinicians and a consensus workshop, the programme will consist of stability and postural correction exercises, general mobility, therapeutic strengthening and stretching exercises supported by an individualised and written exercise sheet that changes over time in line with exercise progression, using PhysioTools, a computer package. The exercise programme will comprise three stages and aim to support the patient back to their specific everyday physical activities including sporting activities where relevant.

Stage one will include postural correction, scapular stability control and proprioceptive exercises. Stage two will progress to range of movement exercises with scapular control in pain-free range, including for example shoulder joint forward flexion, abduction and rotation with progression from assisted to full active exercise. In stage two, stretches will be added if required and resistance muscle strengthening commenced. Stage three will progress the resistance through range to encourage rotator cuff muscle strengthening through all ranges of movement including overhead activity, adding resistance through the use of Theraband (elastic exercise bands) and simple weights. Therapists will encourage exercise behaviour using approaches such as self-monitoring and goal setting. These plans meet recommendations [4] that resistance exercise should be avoided for 2 weeks following steroid injection.

Participants who fail to attend for any physiotherapy appointment will be offered up to two further appointments. After failing to attend the third appointment, no further appointments will be offered.

Attendance at the physiotherapy-led individualised, supervised and progressed exercise sessions will be monitored, and all non-attended sessions will be recorded on the appropriate case report form. The frequency and duration of home exercise will be assessed in self-complete follow-up questionnaires.

#### Advice and exercise leaflet

Clinicians in the interface service shoulder clinics will provide the advice and exercise leaflet following the injection. The leaflet includes information about shoulder anatomy and SIS, plus simple self-help messages about pain relief (including the application of cold packs) and activities. It includes a small number of standardised exercises, including specific muscle strengthening and range of motion exercises [4]. Exercises are not individualised, supervised or progressed by physiotherapists.

### Training of participating interface clinicians and physiotherapists

Prior to recruitment of participants into the trial, all interface clinicians and physiotherapists delivering treatment will be trained to deliver the trial interventions according to the trial intervention protocols and to record the interventions on standardised case report forms.

Interface clinicians will attend a half-day group training session prior to the study commencing which will include training regarding study procedures, eligibility assessment, protocols for US-guided or blind injections, and the delivery of the advice and exercise leaflet. Further refresher sessions will be provided during the course of the trial.

Interface clinicians performing blind (unguided) injections will have extensive clinical experience performing subacromial injections. Since musculoskeletal ultrasound is highly operator-dependent and relies on robust training with direct supervision to gain clinical competency, interface clinicians performing US-guided injections will either have extensive clinical experience performing US-guided injections or be required to complete a Consortium for the Accreditation of Sonographic Education (CASE) [40] accredited course with focus on US-guided subacromial injections. Furthermore, all US-guided injectors will be required to pass the same tests of clinical competency in US-guided subacromial injections assessed by a Consultant Musculoskeletal Sonographer prior to commencement of the study.

Physiotherapists will attend a two-day group training session prior to the trial commencing which will cover information regarding trial procedures, anatomy and physiology relevant to SIS, current best practice guidelines [4], treatment options, and the detailed protocol for individualising, supervising and progressing exercises. A written manual of instructions on how to deliver the interventions will be given to each physiotherapist. Further refresher sessions will be provided during the course of the trial.

Treatment sessions will be audited at regular intervals throughout the trial through case report forms completed by both interface clinicians and physiotherapists at clinic, case-note reviews and observation of physiotherapy sessions by a member of the research team to ensure the interventions are delivered in accordance with the intervention protocols. Video recordings of US-guided injections will be taken throughout the study for audit and training purposes.

### Potential (serious) adverse events

The occurrence of all potential adverse events from all interventions will be monitored and assessed, using case report forms, contact with the trial co-ordinator, family practitioner report, and follow-up questionnaires. If patients suspect that they have suffered an adverse event as a result of any of the trial interventions, they will be asked to notify the trial co-ordinator by telephone, and to seek medical advice from their family practitioner. In our letter informing participants’ family practitioners of trial participation, we will ask the family practitioner to notify the trial co-ordinator if their patient sustains either an adverse or serious adverse event as a result of this trial.

There are usually very few side-effects from corticosteroid injections, delivered either unguided (blind) or with the use of US-guidance. [14,20,27-29]. The interface clinician performing the injection will advise the patient how to manage any potential symptoms due to potential adverse events.

A common adverse event from the exercise regime is expected to be symptoms of temporary, mild soreness of the shoulder joint or upper limb. Participants randomised to the physiotherapist-delivered exercise programme will be advised how to manage such symptoms by the treating physiotherapist. Participants randomised to receive the Advice and Exercise Leaflet will be able to find similar information in the leaflet.

### Follow-up

Outcome measures will be collected before randomisation and at 1 week, 6 weeks, 6 months and 12 months (Table 1).

Participants will be contacted by a research nurse blind to treatment allocation 1 week after randomisation by text-message or telephone according to patient preference. Participants will be asked whether they still have shoulder pain (yes/no) and to rate their current shoulder pain intensity (0-10 numeric rating scale (NRS)). Non-responders to the initial text-message/telephone call will be contacted again 24 hours later. Those who do not respond to this reminder will be contacted by telephone a further 24 hours later. Data will be transposed by the research nurse onto a standard case report form.

Outcome at 6 weeks, 6 months and 12 months will be collected by postal questionnaire (Table 1). Non-responders to follow-up questionnaires will be sent a reminder postcard after two weeks. Those who do not respond to the reminder postcard will be sent a repeat questionnaire and Participant Information Sheet with a further covering letter four weeks after the initial mailing. Non-responders to the repeat questionnaire will be telephoned by the Research Nurse (who will remain blind to group allocation), in order to try to capture key primary outcome data and to minimise missing data. A postal minimum data collection form will be mailed to the participant if the participant has not been contacted after 5 phone-call attempts or if 2 weeks have lapsed from the mail-out of the reminder letter and follow-up questionnaire.

### Outcome measures

Content of trial questionnaires is shown in Table 1. The primary outcome measure is the Shoulder Pain and Disability Index (SPADI) [32]. Secondary outcomes include current shoulder pain intensity (0-10 NRS), patient’s self-reported global impression of change [20], general health [38], sleep [33], pain self-efficacy [35], fear-avoidance [36], return to desired activities including work and social life, work performance [39], healthcare utilisation (medications, visits to healthcare practitioners, further injections or physiotherapy), health status [37], treatment preferences and expectations, illness perceptions [34], exercise adherence and satisfaction with treatment. Potential adverse events from all interventions will be monitored.

### Sample size

The trial is powered, at the 80% level, to detect a ‘small’ to ‘moderate’ effect size (standardised mean difference, 0.4) in shoulder pain and disability (SPADI) for the two main effects (US-guided injection versus blind injection; individualised, progressed and supervised exercise versus standardised advice and exercise leaflet). To address whether the physiotherapy-led intervention consisting of individualised, supervised and progressed exercise provides better improvements in pain and function than a standardised advice and exercise leaflet, we will use the total SPADI score at the 6 month time-point, as the primary outcome measure. Since previous trials have shown that corticosteroid injections improve pain and function more rapidly than exercise [14,20,21], we will use the total SPADI score at the 6 week time-point as the primary outcome measure for the comparison of US-guided and unguided (blind) injection.

A total sample size of 250 participants is needed given 80% power; 5% two-tailed significance; and assuming 20% loss to follow up.

### Analysis

The primary intention-to-treat (ITT) analysis will be double-analysed independently by two blinded statisticians. Between-group analysis will be undertaken according to randomised allocation with retention of all participants throughout follow up for the purposes of full evaluation. The primary evaluations will be mean differences in total SPADI scores at 6 weeks between those randomised to receive US-guided injection versus unguided injection, and mean differences in total SPADI scores at 6 months between those randomised to physiotherapist-led individualised, supervised and progressed exercise versus advice and exercise leaflet. Secondary analyses will include comparison of pain and disability subscales of the SPADI; long-term SPADI outcomes (i.e. at 12 months); and analysis of other outcome measures. Estimates of clinical effect of the two main comparisons will be shown as percentage differences (for dichotomous outcomes) and mean differences (for numerical outcomes), with 95% confidence intervals. P-values will be given alongside statistical testing of the data. Multilevel longitudinal (mixed-) model regression methods (linear for numerical outcomes and logistic for dichotomous outcomes) will be used to generate estimates of effects. By including the baseline values as outcome the model retains all available data (as opposed to complete-case data only) and upholds the full intention-to-treat analytic criterion. An assumption of this approach is that the missing data is missing at random (MAR) – being dependent on baseline factors utilised in the model. Covariates included in the regression models are as follows: age, gender, baseline SPADI scores, duration of pain and location of clinic. Sensitivity analyses will include any other variables which are unbalanced between the groups at baseline.

Primary interest focuses on main effects (‘at the margins’) evaluation as our assumption is that the two interventions will act largely independently of each other. As a secondary analysis, we will estimate the interaction effect from a separate regression model (inclusive of interaction term) since any potential synergistic/antagonistic effect of the combination of treatments should be reported [41]. Descriptively, we will also present mean SPADI scores (95% CIs) for each of the four treatment groups. A sizeable interaction (contrary to expectation) would imply that findings based on an analysis of main effects alone are not sufficient as an effect of combined treatments may exist and should be considered alongside policy recommendations for the two treatments.

A per protocol analysis (analysing only those patients who received the interventions in line with trial protocol) will be carried out as a sensitivity analysis.

### Economic evaluation

Economic evaluation will estimate the cost-effectiveness of the trial interventions. A cost-consequence analysis will initially be reported, describing all the important results relating to costs and consequences in the intervention arms over the 12-month trial period. Subsequently, an incremental cost-utility analysis will be undertaken using patient responses to the EQ-5D-5L, to calculate the cost per additional Quality-Adjusted Life Year (QALY) gained over the same period. The analysis will adopt a broad perspective, considering NHS, patient and productivity costs. Data on health care resource use and time off work over 12 months follow-up will be obtained from the patient questionnaires. The resource use questions will capture details covering a broad range of health care resources, including prescribed and over-the-counter purchased medications, primary care and secondary care (inpatient and outpatient) attendances, treatments and investigations. Unit costs will be obtained from standard sources and health care providers, including NHS reference costs and the British National Formulary. Owing to the paucity of high-quality unit cost data for private health care consultations, these data will be costed as the NHS equivalent. Indirect costs, over 12 months follow-up, will be based on productivity losses due to work incapacity linked to presenteeism, and also work absenteeism. Costs for productivity loss will be computed through the product of lost productivity time (whether presenteeism and/or absenteeism) and the mean hourly wage of patients classified from data on wages corresponding to self-reported baseline occupation (derived from the Annual Surveys of Health Evaluation by the Office of National Statistics and based on classification codes under the UK Socio-Economic Occupation Classification, SOC 2000) QALYs will be calculated using the EQ-5D 5L index scores at baseline, 6 weeks, 6 months and 12 months, using the area-under-the-curve method.

There is no consensus regarding how economic evaluations should be carried out alongside factorial trials and previously published economic evaluations alongside factorial trials have used two main methods: the ‘within the table’ approach [42] and the ‘at the margins’ approach [43]. The base-case economic analysis here will use the ‘at the margins’ approach and follow the primary objectives of the clinical trial. Therefore two separate analyses will be undertaken to consider (1) the physiotherapist–led exercise intervention versus a standardised advice and exercise leaflet and (2) US-guided subacromial injection versus blind injection. Two secondary analyses will also be undertaken. The first will mirror the secondary analysis of the trial and using regression modelling, explore the impact on the results of inclusion of an interaction term. The second will conduct a ‘within the table’ analysis and consider all four treatment options. This method requires interventions to be ordered in terms of increasing cost, and cost and outcomes for each intervention are compared incrementally. The most cost-effective option will be selected based on the principles of simple (strong) dominance (where an intervention is less costly and more effective) and extended (weak) dominance (where an intervention is ruled out if the ICER is greater than that of a more effective intervention).

Missing cost and utility data will be imputed using multiple imputation through chained equations, with the number of imputation datasets set to reflect the proportion of missingness in the data as recommended by previous guidelines into the use of MI approaches [44]. All analyses will be adjusted for baseline EQ-5D 5L score. The comparison results will be presented using cost-effectiveness planes and cost-effectiveness acceptability curves, generated using bootstrap techniques, to reflect sampling variation. Sensitivity analyses (both simple and probabilistic) will be conducted as necessary to explore the importance of key uncertainties in any assumptions made in the base case analysis.

## Discussion

The SUPPORT trial will compare the clinical and cost-effectiveness of (1) US-guided corticosteroid injection to unguided (blind) corticosteroid injection and (2) a physiotherapist-delivered individualised, supervised and progressed exercise programme to provision of a standardised advice and exercise leaflet, for treatment of patients with SIS. In comparison to existing trials of injection and exercise interventions for SIS, the strengths of the SUPPORT trial are its size, long-term follow-up and inclusion of an individualised, supervised and progressed exercise programme which builds upon existing guidelines [4] to optimise treatment outcome [12-15] and inclusion of a cost-effectiveness analysis. The main limitation, common to many trials of non-pharmacological interventions, is the inability to blind participants to treatment allocation. Whilst existing trials suggest some efficacy of both corticosteroid injections and exercises to treat SIS [10,16], the findings of the SUPPORT trial will inform clinical practice about optimal delivery of these interventions to improve pain and function in primary care and at the primary-secondary care interface.

## Abbreviations

GP: General practitioner; GPwSI: General practitioner with a special interest; MSUS: Musculoskeletal ultrasound; NHS: National Health Service; NRS: Numeric rating scale; SIS: Subacromial impingement syndrome; US-guided: Ultrasound-guided.

## Competing interests

The authors declare that they have no competing interests.

## Authors’ contributions

All authors participated in the design of the trial and the drafting of the manuscript. All authors have read and approved the final manuscript.

## Pre-publication history

The pre-publication history for this paper can be accessed here:

http://www.biomedcentral.com/1471-2474/15/81/prepub
